# α-Amylase and dipeptidyl peptidase-4 (DPP-4) inhibitory effects of *Melicope latifolia* bark extracts and identification of bioactive constituents using *in vitro* and *in silico* approaches

**DOI:** 10.1080/13880209.2021.1948065

**Published:** 2021-08-04

**Authors:** Alexandra Quek, Nur Kartinee Kassim, Pei Cee Lim, Dai Chuan Tan, Muhammad Alif Mohammad Latif, Amin Ismail, Khozirah Shaari, Khalijah Awang

**Affiliations:** a Department of Chemistry, Faculty of Science, Universiti Putra Malaysia, UPM Serdang, Selangor, Malaysia; b Integrated Chemical BioPhysics Research, Faculty of Science, Universiti Putra Malaysia, UPM Serdang, Selangor, Malaysia; c Faculty of Pharmacy, Mahsa University, Bandar Saujana Putra, Jenjarom, Selangor, Malaysia; d Department of Nutrition and Dietetics, Faculty of Medicine and Health Sciences, Universiti Putra Malaysia, UPM Serdang, Selangor, Malaysia; e Natural Medicines & Products Research Laboratory, Institute of Bioscience, Universiti Putra Malaysia, UPM Serdang, Selangor, Malaysia; f Department of Chemistry, Faculty of Science, Universiti Malaya, Kuala Lumpur, Malaysia

**Keywords:** Diabetes, antidiabetic, molecular docking, antioxidant

## Abstract

**Context:**

*Melicope latifolia* (DC.) T. G. Hartley (Rutaceae) was reported to contain various phytochemicals including coumarins, flavonoids, and acetophenones.

**Objective:**

This study investigates the antidiabetic and antioxidant effects of *M. latifolia* bark extracts, fractions, and isolated constituents.

**Materials and methods:**

*Melicope latifolia* extracts (hexane, chloroform, and methanol), fractions, and isolated constituents with varying concentrations (0.078–10 mg/mL) were subjected to *in vitro* α-amylase and dipeptidyl peptidase-4 (DPP-4) inhibitory assay. Molecular docking was performed to study the binding mechanism of active compounds towards α-amylase and DPP-4 enzymes. The antioxidant activity of *M. latifolia* fractions and compounds were determined by 2,2-diphenyl-1-picrylhydrazyl (DPPH) free radical scavenging and β-carotene bleaching assays.

**Results:**

*Melicope latifolia* chloroform extract showed the highest antidiabetic activity (α-amylase IC_50_: 1464.32 μg/mL; DPP-4 IC_50_: 221.58 μg/mL). Fractionation of chloroform extract yielded four major fractions (CF_1_–CF_4_) whereby CF_3_ showed the highest antidiabetic activity (α-amylase IC_50_: 397.68 μg/mL; DPP-4 IC_50_: 37.16 μg/mL) and resulted in β-sitosterol (**1**), halfordin (**2**), methyl *p*-coumarate (**3**), and protocatechuic acid (**4**). Isolation of compounds **2**–**4** from the species and their DPP-4 inhibitory were reported for the first time. Compound **2** showed the highest α-amylase (IC_50_: 197.53 μM) and β-carotene (88.48%) inhibition, and formed the highest number of molecular interactions with critical amino acid residues of α-amylase. The highest DPP-4 inhibition was exhibited by compound **3** (IC_50_: 911.44 μM).

**Discussion and conclusions:**

The *in vitro* and *in silico* analyses indicated the potential of *M. latifolia* as an alternative source of α-amylase and DPP-4 inhibitors. Further pharmacological studies on the compounds are recommended.

## Introduction

Diabetes mellitus is a chronic metabolic condition characterized by elevated blood glucose levels caused by insulin deficiency and/or ineffective insulin action. In 2019, 463 million people were affected by diabetes mellitus and the number of diabetic cases is expected to hit 700 million in 2045 (Saeedi et al. 2019). Diabetes mellitus has been associated with various complications including retinopathy, nephropathy, neuropathy, peripheral artery disease, cardiovascular disease, and stroke (Rangel et al. 2019). Current therapeutic approach against diabetes mellitus includes insulin injections and oral hypoglycaemic drugs such as metformin, acarbose, sitagliptin, and glimepride. However, many clinical hypoglycaemic drugs have been reported to cause unfavourable side effects on the recipient’s health. For instance, pancreatitis, congestive heart failure, gastrointestinal discomforts, and diarrhoea are among the side effects experienced by consumers (Akhtar et al. 2018; Kalhotra et al. 2019). These side effects along with the high cost of clinical drugs have led to the diabetic patients’ poor adherence to hypoglycaemic drugs (Polonsky and Henry 2016). Therefore, research on alternative treatment for diabetes mellitus from natural sources have increased tremendously in recent years.

Several studies have made suggestions of oxidative stress being a common factor in the functional damage of pancreatic β-cells. The β-cells are sensitive to reactive oxygen species (ROS) due to their low antioxidant enzymes count, and, hence, are susceptible to ROS-induced damage (Wang and Wang 2017). Moreover, there is strong evidence towards the association of diabetic-induced oxidative stress with the development of diabetes complications. For instance, inflammation, cell death, mitochondrial dysfunction, and neurodegeneration caused by oxidative stress leads to retinal, neural, and vascular tissue damage (Olvera-Montaño et al. 2019). Accordingly, plants with antioxidant constituents that can scavenge the ROS should be considered as alternative leads in diabetes prevention and management.

Natural products, especially those that are plant-based, play a vital role as the primary quarry for finding promising lead candidates in prospective drug development programs. A plant-based traditional medicinal system is mainly practiced in developing countries that possess high plant biodiversity, and acts as the primary healthcare support of about 75–80% of the world population (Tran et al. 2020). Developing countries regard plant-based medications as their primary choice of treatment due to their ease of availability, lesser side effects, and relative cultural familiarity and acceptance compared to chemically synthesized drugs (Salehi et al. 2019). In recent years, many medicinal plants have been known to be effective in diabetes management with more than 400 species reported to exhibit antihyperglycemic activities (Malviya 2010). To date, the search for new antidiabetic therapies from plants is still of great interest due to the presence of phytochemical compounds in plants which can serve as an alternative and safer treatment for diabetes mellitus.

*Melicope*, the largest genus of Rutaceae, with approximately 235 species identified has been widely adopted in the traditional treatment of various human maladies including hypertension, fever (Nguyen et al. 2016), stomach-ache, rheumatism (Sulaiman et al. 2010), and diabetes (Eliaser et al. 2018). *Melicope latifolia* (DC.) T. G. Hartley is a wild evergreen shrub that naturally occurs in Malaysia, Indonesia, the Philippines, and Papua New Guinea. In Malaysia, the plant is commonly known as ‘Ki sampang’ or ‘Pepau’, while Indonesian folklore often associates the usage of the plant with the relief of cramps and fever. Phytochemical studies have reported the presence of flavonoids, coumarins, alkaloids, acetophenones, and terpenoids in *M. latifolia* (Susiloningrum et al. 2020).

Through the years, various phenolic compounds have been regarded as effective antioxidants and robust in the treatment of diabetes mellitus. Esculetin, umbelliferone, bergapten, emodin, and kaempferol are examples of plant phenolics that were previously described to regulate hyperglycaemia (Lin et al. 2019). Despite the potential, studies on antidiabetic properties of *M. latifolia* have yet to be reported. Thus, in this work, α-amylase and DPP-4 inhibitory activities of different extracts, fractions, and isolated compounds from the bark of *M. latifolia* were evaluated using *in vitro* assays. *In silico* molecular docking was used to study the binding affinity and interactions between the active compounds and the enzymes. The antioxidant activity of the fractions and isolated constituents were also investigated. Herein, we describe for the first time how *M. latifolia* exhibits inhibitory actions against α-amylase and DPP-4. The isolation of halfordin (**2**), methyl *p*-coumarate (**3**), and protocatechuic acid (**4**) from *M. latifolia* and their DPP-4 inhibitory activities are also reported for the first time in this study.

## Materials and methods

### Chemicals and reagents

Column chromatography (CC) was carried out using silica gel Merck Kieselgel PF254 Art. No. 1.07734.1000 and silica gel Merck Kieselgel PF254 Art. No. 9385.1000. Thin layer chromatography (TLC) was performed using Merck DC-Plasticfolie TLC plastic sheet pre-coated with Kiecelgel 60 PF (Merck, Kenilworth, NJ). Acarbose, dimethylsulphoxide (DMSO), ascorbic acid, α-tocopherol, butylated hydroxytoluene (BHT), hexane, ethyl acetate (EtOAc), chloroform (CHCl_3_), methanol (MeOH), and deuterated solvents (chloroform and acetone) were purchased from Sigma Aldrich (St. Louis, MO). α-Amylase was purchased from Megazyme (Co. Wicklow, Ireland). DPP-4 inhibitory screening kit was purchased from Cayman Chemical (Ann Arbor, MI).

### Plant materials

The bark of *M. latifolia* was collected from Bukit Serting, Negeri Sembilan, Malaysia, in 2008. The plant was identified by Mr. Teo Leong Eng. A voucher specimen (No. KL 5538) was deposited at the Department of Chemistry, Universiti of Malaya, Malaysia.

### Preparation of crude extracts

Shade-dried bark of *M. latifolia* (1.0 kg) was pulverized to obtain a fine powder. The fine powder was macerated with hexane (5 L) for over 72 h at ambient temperature and subsequently filtered. The residue was re-extracted twice with a fresh batch of hexane. The filtrates were combined and dried with a rotary evaporator at 40 °C to obtain a crude hexane extract. The residues were then macerated again successively with chloroform and followed by methanol in the same manner to acquire chloroform and methanol crude extracts. The hexane extract was obtained as a dark brown gummy form while chloroform and methanol extracts were brown solid and orange-brown gummy in form, respectively. The mass of all the extracts were recorded and subjected to antidiabetic and antioxidant assays. An assay guided isolation technique based on antidiabetic assays of α-amylase and DPP-4 inhibitory assays was applied in an attempt to isolate the bioactive constituents.

### Determination of extraction yield

The extraction yield (%) of *M. latifolia* was calculated as previously described by Truong et al. (2019) using the following formula:

(1) Extraction yield (%)=[(dry weight of extract)/(dry weight of sample)]×100

### Assay-guided isolation of chemical constituents from *Melicope latifolia*

The separation of the chloroform extract of *M. latifolia* (17.0 g) was conducted using column chromatrography (CC) (8.5 × 20.0 cm) over silica gel 60 (70–230 mesh) and eluted with the increasing polarity of solvent systems starting with hexane, hexane:EtOAc, EtOAc:MeOH, and MeOH. A total of 128 fractions (F1–F128) of 200 mL each were collected from the column chromatography. The column fractions were then pooled based on their thin-layer chromatography (TLC) profiles into four major fractions, CF_1_ (F1–F34), CF_2_ (F35–F62), CF_3_ (F63–80) and CF_4_ (F81–128). The most active fraction with potent enzymes inhibitory activity, CF_3_ (5.3 g) was further chromatographed over silica gel 60 (230–400 mesh) and eluted with increasing polarities of solvent systems starting from hexane:EtOAc (4:1), EtOAc:MeOH to MeOH and gave 11 sub-fractions, CF_3.1_–CF_3.11_. Purification of CF_3.2_ (41.0 mg) by washing with hexane gave white crystals β-sitosterol (**1**) (12.0 mg). CF_3.7_ (74.0 mg) was subjected to chromatograph over silica gel 60 (230–400 mesh) with the solvent system hexane:acetone (4.5:0.5) to obtain 20 sub-fractions, CF_3.7.1_–CF_3.7.20_ of 50 mL each. From the 20 sub-fractions, colourless needles halfordin (**2**) (6.4 mg) and methyl *p*-coumarate (**3**) (5.1 mg) were successfully yielded from CF_3.7.3_ and CF_3.7.16,_ respectively. CF_3.11_ (30.0 mg) was purified by using Sephadex LH-20 with CHCl_3_:MeOH (1:1) to yield colourless needle protocatechuic acid (**4**) (3.4 mg).

### Structure elucidation of isolated constituents

A combination of nuclear magnetic resonance (NMR) spectroscopy (JEOL 500 MHz NMR), mass spectrometry (JEOL Ltd., Tokyo, Japan), and FT-IR were used to identify the isolated compounds. Tetramethyl silane (TMS) was used as an internal standard for NMR analysis. Mass spectral data of the pure compounds were obtained using a Shimadzu QP 5050 A Spectrophotometer (Shimadzu Corporation, Kyoto, Japan) with a direct injection probe. One-dimensional (1D) (^1^H, ^13^C) and two-dimensional (2D) (COSY, HMQC, HMBC, DEPT) NMR spectra of pure compounds were processed using MestReNova software ver. 14.1.2 (Mestrelab Research S.L., Santiago de Compostela, Spain). The structures of the isolated compounds were confirmed by comparing their spectroscopic data with literature data.

### Determination of antidiabetic activities

#### α-Amylase inhibitory assay

The α-amylase inhibition assay was conducted by adapting a method described by Abdullah and Kasim (2017) with slight modifications. Briefly, 60 μL of 0.1 M sodium phosphate buffer (pH 6.9) was mixed with test samples (30 μL) of varying concentrations (0.078-10 mg/mL) in a 96-well microplate. 10 μL of α-amylase (1 U/mL) was then added into the wells. The plate was subjected to incubation for 15 min at 37 °C before adding 30 μL of soluble starch (1.0%). After another 30 min of incubation at 37 °C, the reaction in the mixture was halted by adding hydrochloric acid (30 μL) followed by the addition of iodine regent (30 μL). The absorbance of the mixture was taken at a wavelength of 620 nm. Phosphate buffer and acarbose were used to replace the test samples as negative and positive controls, respectively. The α-amylase inhibition activity was calculated using the formula (2) as follows:

(2) $$\text{\% inhibition }=\;[({\mathrm{OD}}_{\mathrm{sample}}\;–\;{\mathrm{OD}}_{\mathrm{control}})/({\mathrm{OD}}_{\mathrm{sample}})]\times 100\%$$

where OD_sample_ is the absorbance of sample; OD_control_ is the absorbance of negative control.

### DPP-4 inhibitory assay

DPP-4 inhibitor screening kit (Cayman Chemical, Ann Arbor, MI) was used to evaluate the DPP-4 inhibitory activity of the test samples based on a fluorescence assay method. Briefly, samples (10 μL) with different concentrations in DMSO were each pipetted into a 96-well plate followed by the addition of diluted assay buffer (30 μL), diluted human-recombinant DPP-4 enzyme solution (10 μL), and diluted fluorogenic substrate, Gly-Pro-aminomethylcoumarin (AMC) (50 μL). For the negative and positive control wells, the sample was replaced by a solvent and sitagliptin standard, respectively. The 96-well plate was shaken and incubated for 30 min at 37 °C. After incubation, the fluorescence of the free AMC group resulting from the reaction was monitored on an excitation wavelength range of 350–360 nm and an emission wavelength range of 450–465 nm using a microplate reader. The percentage of inhibition was calculated using the formula (3):

(3) $$\text{\% inhibition}=[({\mathrm{OD}}_{\text{control }}-\;{\mathrm{OD}}_{\mathrm{sample}})/({\mathrm{OD}}_{\mathrm{control}})]\times 100\%$$

whereby OD_control_ is the absorbance of negative control; OD_sample_ is the absorbance of sample.

### *In silico* molecular docking

To comprehend the binding mechanism of the active compounds with α-amylase and DPP-4, molecular docking was performed using the X-ray crystal structure of human pancreatic α-amylase complexed with acarbose derived pentasaccharide (PDB ID 3BAJ) (Nazir et al. 2018) and human DPP-4 complexed with drug sitagliptin (PDB ID 1X70) (Quek et al. 2020), both of which were retrieved from the Protein Data Bank (https://www.rcsb.org/pdb). AutodockTools (ADT) ver. 1.5.6 (Yusof et al. 2019) was used for the preparation of the molecular docking, whereby water molecules, non-polar hydrogens and unwanted chains were removed from the protein structure and polar hydrogens were added. The molecular structures of the ligands were built and optimized using ChemDraw 16.0 software (PerkinElmer Informatics, Waltham, MA). Gasteiger charges were assigned to all the atoms involved in the docking using ADT. Autodock Vina (Quek et al. 2020) was used to dock and predict the binding affinity of all the ligands. The binding site was defined by the binding of reference ligands acarbose derived pentasaccharide and sitagliptin in the X-ray crystal structures of α-amylase and DPP-4, respectively (Kalhotra et al. 2019). The docking was performed inside a 26 × 26 × 26 Å grid box, centred on the binding site of the DPP-4 at (*x* = 40.797, *y* = 50.515, *z* = 35.259) with a grid spacing of 1.0 Å. For α-amylase, the grid box was centred at (*x* = 7.883, *y* = 17.194, *z* = 41.901) employing the same size and grid spacing. For each enzyme–ligand interaction, the pose with the lowest binding affinity was analyzed using Discovery Studio Visualizer software (Abdullahi and Adeniji 2020). The docking parameters were validated by extracting and redocking co-crystallized ligands into their respective crystal enzyme receptor (Shah et al. 2019).

### Determination of antioxidant activities

#### 2,2-Diphenyl-1-picrylhydrazyl (DPPH) free radical scavenging assay

Free radical scavenging ability of *M. latifolia* fractions and compounds were determined using stable free radical 2,2-diphenyl-1-picrylhydrazyl (DPPH) following the method described by Tan et al. (2019). The extracts (170 μL) at varying concentrations (15.6–1000 mg/mL) were mixed with 30 μL of methanolic DPPH solution (300 μM) in a 96-well microplate. The mixtures were then incubated at room temperature for 30 min in the dark, followed by an absorbance measurement at 517 nm. For the negative control well, the extract was replaced by methanol. Ascorbic acid, α-tocopherol, and BHT were used as standards. The percentage of free radical scavenging ability was calculated using the formula (4):

(4) $$\text{\% inhibition}=[({\mathrm{OD}}_{\text{control }}-\;{\mathrm{OD}}_{\mathrm{sample}})/({\mathrm{OD}}_{\mathrm{control}})]\times 100\%$$

whereby OD_sample_ is the absorbance of sample; OD_control_ is the absorbance of negative control.

### β-Carotene bleaching assay

The antioxidant capacity of *M. latifolia* fractions and isolated constituents by β-carotene bleaching was performed according to a method described by Kassim et al. (2019). In brief, 210 µL of β-carotene solution (1 mg/mL in chloroform) was added to Tween 20 (42 µL) and linoleic acid (5 µL) in a round bottom flask. The chloroform was removed by rotary evaporation prior to the addition of 10 µL of distilled water and shaken to form an emulsion. The emulsion (200 µL) was then transferred into each well of a 96-well microplate that contains 50 µL of samples (1 mg/mL). The mixture was incubated at 50 °C in the dark for 2 h, and its absorbance was measured at 470 nm at the initial time (*t* = 0) and every subsequent 30 min for 2 h (*t* = 2). Ascorbic acid, α-tocopherol, and BHT were used as positive controls. Methanol was used to replace the test samples as a negative control. The antioxidant activity (AA) was calculated according to the formula (5):

(5) $$\text{AA\% }=\;1\;-\;[(A_{t=0}\;–\;A_{t=2})/(A_{c=0}\;–\;A_{c=2})]\;\times \;100$$

whereby A_t=0_ and A_t=2_ are the absorbance of the samples measured at 0 and 2 h, respectively; A_c=0_ and A_c=2_ are the absorbance of negative control measured at 0 and 2 h, respectively.

### Statistical analysis

All experiments were carried out in triplicate. The results are expressed as mean ± standard deviation (SD) and differences between means were statistically analyzed using the *t*-test for comparison between two treatments, with *p* < 0.05 considered significant. The statistical analysis was performed with GraphPad Prism (GraphPad Software, San Diego, CA).

## Results

### Extraction yield

In this study, three different solvents (hexane, chloroform, and methanol) were used in the order of increasing polarity for the sequential extraction of *M. latifolia*. Different solvents were used to target the extraction of non-polar, semi-polar, and highly polar compounds. The yield of extraction for hexane, chloroform, and methanolic crude of *M. latifolia* was 0.54%, 1.8%, and 2.1%, respectively (Table 1).

**Table 1.** Extraction yield of various *M. latifolia* extracts.

Plant part	Extracts	Extraction yield (%)
Stem bark	Hexane	0.54 ± 0.11^a^
	Chloroform	1.80 ± 0.03^b^
	Methanol	2.10 ± 0.19^c^

All values were expressed as mean ± standard deviation of triplicates. Data with different superscript (a, b, c) were considered significant (*p* < 0.05).

### Identification of isolated constituents

**Figure 1.**
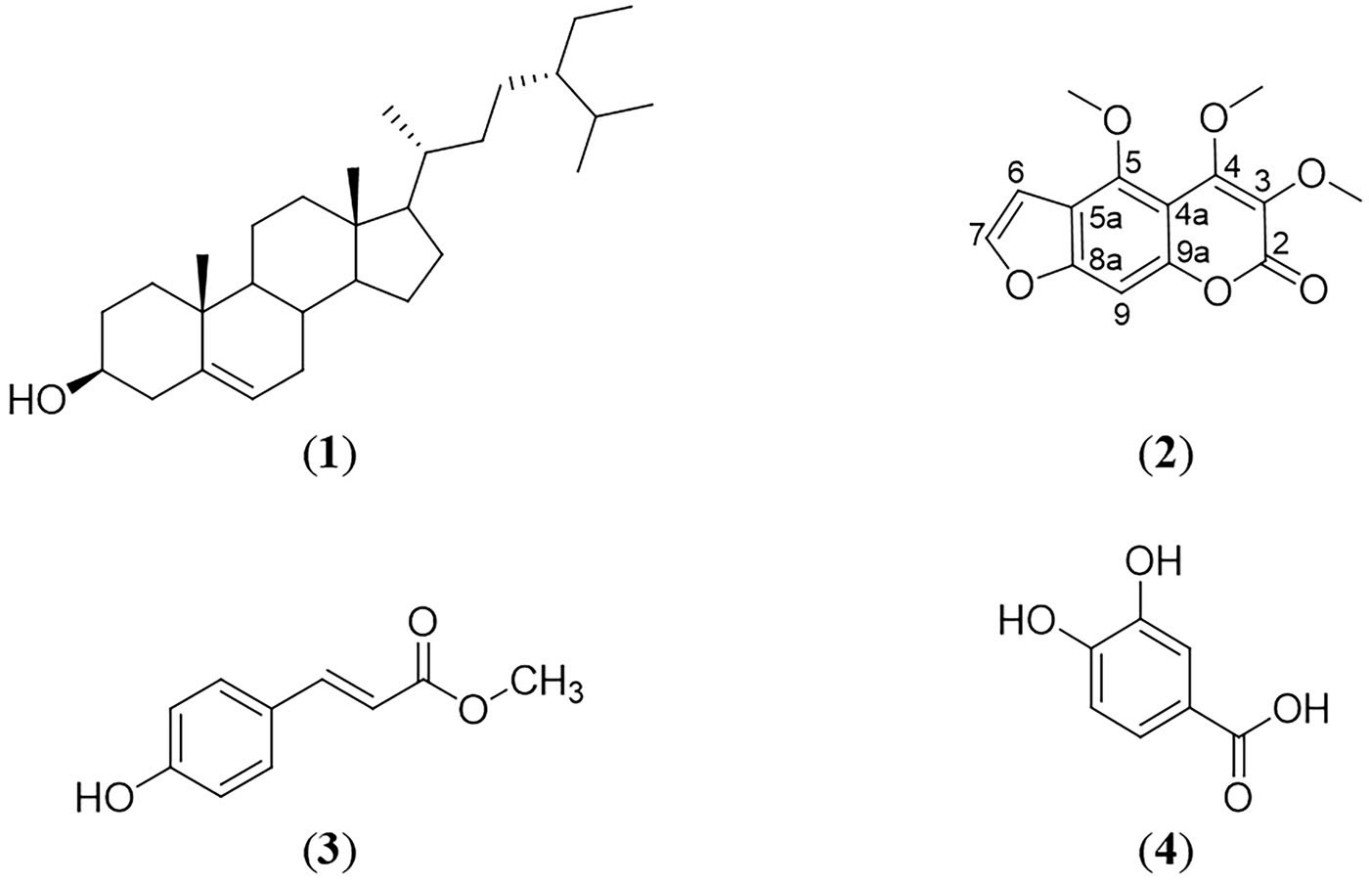
Chemical structures of β-sitosterol (**1**), halfordin (**2**), methyl *p*-coumarate (**3**), and protocatechuic acid (**4**).

Repeated chromatography on the most active fraction, CF_3_ of the chloroform extract successfully led to the isolation of four compounds: β-sitosterol (**1**), halfordin (**2**), methyl *p*-coumarate (**3**), and protocatechuic acid (**4**). 1D and 2D NMR along with EI-MS of the isolated compounds were found to be in agreement with literature, effectively confirming their identification. Compounds **3** and **4** were first isolated from the species whereas compound **2** was first isolated from the genus (Figure 1). NMR and EI-MS spectra of **1**–**4** can be found in Supplemental Figures S1–S4.

Compound **2** was known as furanocoumarin, halfordin based on the comparison of ^1^H and ^13^C NMR data with literature (Sultana et al. 2000). The compound was isolated as colourless needles. Its molecular formula of C_14_H_12_O_6_ was established by EI-MS with a molecular ion [M^+^] peak at *m/z* 276 (calculated for C_14_H_12_O_6_, 276.0634). The ^1^H NMR spectrum of **2** revealed the presence of three methoxy groups at *δ* 3.91 (3-OCH_3_), *δ* 4.22 (4-OCH_3_), and *δ* 4.03 (5-OCH_3_). A pair of doublet resonances sharing the same coupling constant at *δ* 7.58 (1H, *J* = 2.4 Hz) and 6.92 (1H, *J* = 2.4, 1.1 Hz) represent the characteristic peaks of α and β furan protons, H-7 and H-6, respectively. The signal for the unsubstituted proton of the coumarin benzene ring, H-9 was observed as a doublet at *δ* 7.23 (1H, *J* = 1.1 Hz) due to its long-range coupling with H-6, which is typically observed for linear furanocoumarin (Baek et al. 2000). The ^13^C spectrum of **2** further supported the presence of methine carbons of furan ring, C-6 and C-7 at *δ* 104.5 and 145.4, respectively. Meanwhile, the carbon signal at *δ* 160.4 typically represented the carbonyl carbon, C-2 of the lactone ring in **2**.

### Antidiabetic activities

α-Amylase and DPP-4 inhibitory effect of all the *M. latifolia* extracts were recorded as half-maximal inhibitory concentration (IC_50_) and shown in Table 2. The chloroform extract showed the highest inhibitory strength against both α-amylase and DPP-4 enzymes with IC_50_ values of 1464.32 and 221.58 μg/mL, respectively. Meanwhile, the lowest inhibitory potency against the enzymes can be observed in the hexane extract with IC_50_ values of 8113.15 μg/mL against α-amylase and 5872.03 μg/mL against DPP-4. The inhibitory potency of *M. latifolia* extracts against α-amylase and DPP-4 in descending order was chloroform > methanol > hexane, thus, justifying the further fractionation of the chloroform extract. The chloroform extract was fractionated using a gradient solvent system consisting of hexane, ethyl acetate, and methanol to obtain four major fractions, CF_1_–CF_4_.

**Table 2.** α-Amylase and DPP-4 inhibitory activities of various *M. latifolia* extracts.

Extracts	IC_50_ (μg/mL)	
	α-Amylase	DPP-4
Hexane	8113.15 ± 103.15^c^	5872.03 ± 211.67^c^
Chloroform	1464.32 ± 312.19^a^	221.58 ± 32.01^a^
Methanol	2941.17 ± 113.72^b^	990.21 ± 25.59^b^

All values were expressed as mean ± standard deviation of triplicates. Data with different superscript (a, b, c) in the same column were considered significant (*p* < 0.05).

The inhibitory activities of the α-amylase and DPP-4 of fractions and isolated compounds from *M. latifolia* are presented in Table 3. When tested for α-amylase inhibitory properties, the IC_50_ values of fractions ranged from 397.68 to 1025.41 μg/mL with the most potent inhibition exhibited by fraction CF_3_ and the least by fraction CF_2_. The calculated α-amylase inhibitory IC_50_ values of the fractions in descending order were CF_3_ > CF_4_ > CF_1_ > CF_2_. Fraction CF_3_ also showed the highest DPP-4 inhibition activity with an IC_50_ value of 37.16 μg/mL. The IC_50_ values of all the other fractions against DPP-4 ranged from 80.40 to 581.50 μg/mL. The ranking of DPP-4 inhibitory IC_50_ values of the fractions in descending order were CF_3_ > CF_2_ > CF_4_ > CF_1_. As both the DPP-4 and α-amylase inhibitory assays showed promising results, CF_3_ was further purified in an attempt to identify potential α-amylase and DPP-4 inhibitors.

**Table 3.** α-Amylase and DPP-4 inhibitory activities of *M. latifolia* fractions and isolated compounds.

	IC_50_ (μg/mL)	
	α-Amylase	DPP-4
Fractions		
CF_1_	954.63 ± 32.09^h^	581.50 ± 12.77^d^
CF_2_	1025.41 ± 218.64^i^	80.40 ± 6.84^b^
CF_3_	397.68 ± 10.41^f^	37.16 ± 3.02^a^
CF_4_	831.72 ± 9.25^g^	160.00 ± 15.21^c^
Compounds		
** 1**	154.4 ± 15.80 (372.31 ± 2.62 μM)^c^	ND
** 2**	54.53 ± 3.19 (197.53 ± 4.18 μM)^a^	ND
** 3**	220.60 ± 9.11 (1238.90 ± 22.05 μM)^e^	162.40 ± 1.45 (911.44 ± 7.61 μM)^c^
** 4**	89.31 ± 11.30 (579.94 ± 3.18 μM)^b^	ND
Sitagliptin	–	0.014 ± 0.01 (0.034 ± 0.04 μM)^f^
Acarbose	182.31 ± 20.16 (282.39 ± 8.14 μM)^d^	–

ND: not determined where IC_50_ was not detected at highest tested concentration (1 mg/mL). ‘–’: Not available. All values were expressed as mean ± standard deviation of triplicates. Data with different superscript (a, b, c, d, e, f, g, h, i) in the same column were considered significant (*p* < 0.05).

Among the compounds, compound **2** showed the highest inhibition against α-amylase with an IC_50_ value of 197.53 μM, and was better than the inhibition activity of the positive control, acarbose with an IC_50_ value of 282.39 μM. Compound **1** also showed good inhibitory activity with an IC_50_ value of 372.31 μM. The lowest inhibition effect was observed in compound **3**. The ranking of the α-amylase inhibitory activity of the compounds isolated from CF_3_ based on their IC_50_ values was **2 **>** **acarbose > **1 **>** 4** > **3**. For the DPP-4 inhibition activity, compound **3** showed mild inhibitory activity against DPP-4 with an IC_50_ value of 911.44 μM compared to the positive control sitagliptin, which showed significantly higher inhibitory activity with an IC_50_ value of 0.034 μM. The DPP-4 IC_50_ of compounds **1**, **2**, and **4** were not detected even when tested at the highest concentration of 1 mg/mL. All the compounds were further subjected to *in silico* molecular docking analysis with the α-amylase enzyme, however, only compound **3** was analyzed with DPP-4 enzyme.

### *In silico* molecular docking

**Figure 2.**
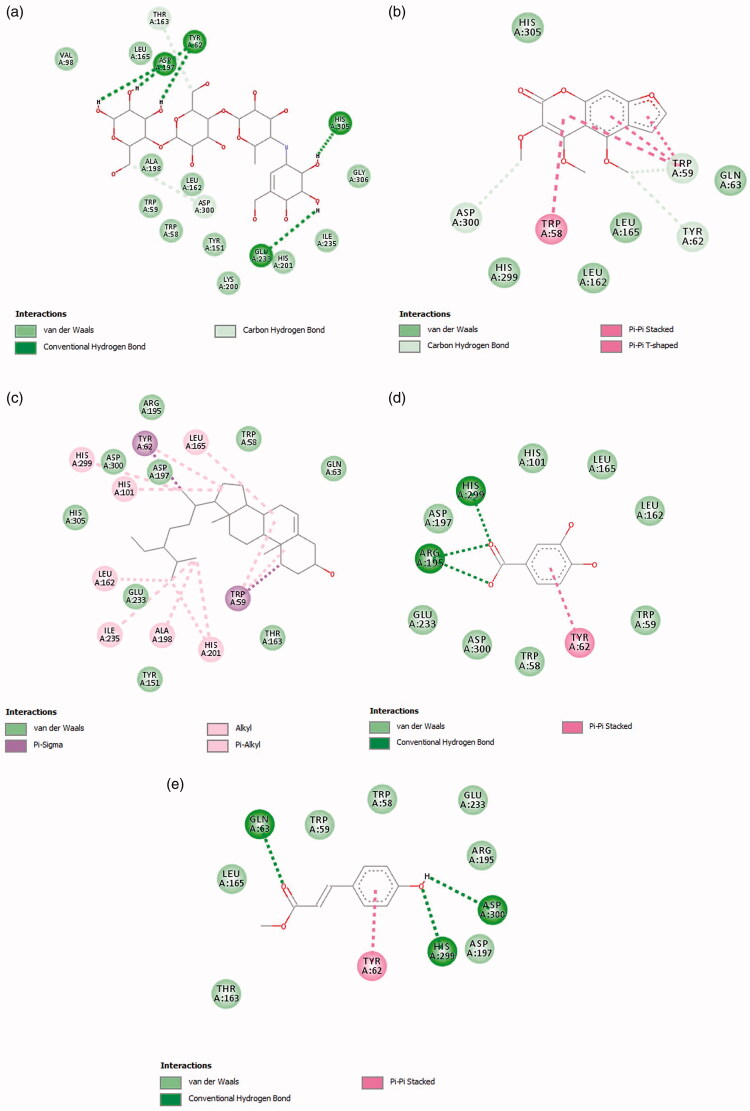
Two-dimensional (2D) interaction diagram of the identified compounds with amino acid residues of α-amylase: (a) acarbose; (b) halfordin, **2**; (c) β-sitosterol, **1**; (d) protocatechuic acid, **4**; (e) methyl *p*-coumarate, **3**.

**Figure 3.**
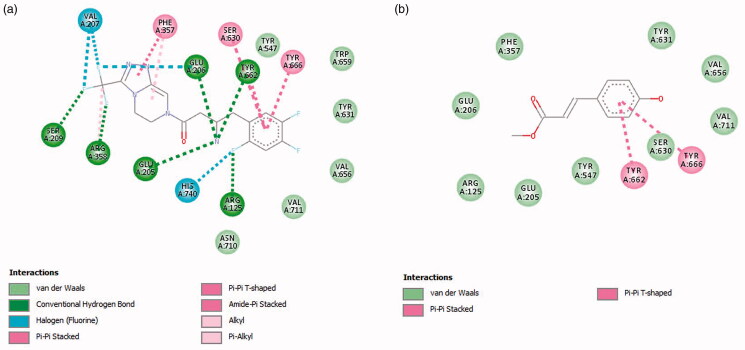
Two-dimensional (2D) interaction diagram of the identified compounds with amino acid residues of DPP-4: (a) sitagliptin and (b) methyl *p*-coumarate, **3**.

*In silico* molecular docking of compounds, **1**–**4** and the standard acarbose was carried out in the active site of α-amylase (PDB ID 3BAJ), while the molecular docking of compound **3** and the standard sitagliptin was performed in the active sites of DPP-4 (PDB ID 1X70). The redocking of co-crystallized ligands (acarbose-derived pentasaccharide and sitagliptin) in their respective enzyme receptors was able to replicate the experimental bindings with root-mean-square deviation (RMSD) values of 1.12 and 0.54 Å, respectively (Supplemental Figure S5). The binding affinity of **1**, **2**, **3**, and **4** with α-amylase were −9.3, −6.6, −5.7, and −6.0 kcal/mol, respectively. A lower or more negative value of the binding affinity indicates a stronger binding interaction between the compound and the enzyme. The complex formed between the standard acarbose and α-amylase gave a binding affinity of −9.1 kcal/mol. Meanwhile, the binding affinity of compound **3** with DPP-4 was −5.7 kcal/mol, which was higher than that of the standard sitagliptin (−8.6 kcal/mol). The 2D binding patterns and docking poses of the compounds with α-amylase and DPP-4 enzymes are shown in Figures 2 and 3, respectively. Their 3D interaction diagrams with the enzymes are shown in Supplemental Figures S6 and S7, and is further summarized in Supplemental Tables S1 and S2.

### Antioxidant activities

The antioxidant activities of the fractions and the isolated constituents from the chloroform extract of *M. latifolia* were evaluated based on their DPPH free radical scavenging and β-carotene bleaching ability (Table 4). Compound **4** was not evaluated for its antioxidant capacity due to an insufficient amount obtained in the experiment. Among the fractions, CF_4_ was found to be the most active in scavenging DPPH radicals with an IC_50_ value of 182.88 μg/mL, followed by CF_1_ with an IC_50_ value of 201.91 μg/mL. The ranking of fractions in retarding DPPH free radicals in descending order were CF_4_ > CF_1_ > CF_3_ > CF_2_. Meanwhile, compound **2** showed mild DPPH scavenging ability with an IC_50_ value of 6429.23 μM whereas the IC_50_ of compounds **1** and **3** were not detected even when tested at the highest concentration (1 mg/mL) due to their very low or absence of radical scavenging activity. Positive controls, namely ascorbic acid, α-tocopherol, and BHT, respectively showed DPPH scavenging effects with their respective IC_50_ values of 85.17, 43.63, and 44.93 μM.

**Table 4.** DPPH-free radical scavenging activity and β-carotene bleaching activity of *M. latifolia* fractions and isolated compounds.

	DPPH	β-Carotene bleaching activity
	IC_50_ (μg/mL)	AA (%)
Fractions		
CF_1_	207.91 ± 0.02^e^	62.87 ± 0.04^d^
CF_2_	601.87 ± 0.04^g^	59.89 ± 0.03^e^
CF_3_	399.03 ± 0.05^f^	58.90 ± 0.06^e^
CF_4_	182.88 ± 0.03^d^	46.43 ± 0.02^f^
Compounds		
** 1**	ND	49.46 ± 0.03^f^
** 2**	1776.01 ± 0.16 (6429.23 ± 0.16 μM)^h^	88.48 ± 0.02^b^
** 3**	ND	1.08 ± 0.02^g^
** 4**	–	–
Ascorbic acid	15.00 ± 0.01 (85.17 ± 0.01 μM)^b^	35.23 ± 0.02^g^
α-Tocopherol	18.79 ± 0.01 (43.63 ± 0.01 μM)^c^	98.01 ± 0.02^a^
BHT	9.90 ± 0.03 (44.93 ± 0.03 μM)^a^	76.42 ± 0.02^c^

ND: Not determined where IC_50_ was not detected at highest tested concentration (1 mg/mL). ‘-’: Not available. All values were expressed as mean ± standard deviation of triplicates. Data with different superscript (a, b, c, d, e, f, g, h) in the same column were considered significant (*p* < 0.05).

For the β-carotene bleaching assay, the antioxidant activity of the fractions assay ranged from 46.43% to 62.87% with the most active fraction exhibited by CF_1_, while the least active being CF_4_. The ranking of the antioxidative actions of the fractions in β-carotene bleaching assay in descending order were CF_1_ > CF_2_ > CF_3_ > CF_4_. Among the compounds, compound **2** showed the highest antioxidant activity (88.48%) which was higher than the antioxidant activity of BHT (76.42%) The ranking of the antioxidant activity in β-carotene bleaching is in the order of α-tocopherol > **2 **>** **BHT > **1 **>** **ascorbic acid > **3**.

## Discussion

In this study, *M. latifolia* extracts inhibited both α-amylase and DPP-4 in which the chloroform extract demonstrated the highest inhibition activities. The finding was in accordance with studies by Geng et al. (2013) and Tan et al. (2019) who reported that the chloroform extracts had the highest inhibitory activities against α-amylase and DPP-4 compared to other solvent crude extracts. This could be attributed to the presence of intermediate polar compounds that are responsible for the inhibition of these enzymes. For instance, flavonoids and coumarins that are rich in middle polar extracts have been previously known as potent α-amylase and DPP-4 inhibitors (Lin et al. 2019). Previous studies have shown that *M. latifolia* extracts exhibited higher α-amylase and DPP-4 inhibitory activities compared to *Salacia oblonga* Wall. (Celastraceae) and *Rubus caesius* L. (Rosaceae) extracts, respectively (Divya et al. 2014; Chelladurai and Chinnachamy 2018), however, the α-amylase and DPP-4 inhibitory activities of *Kappaphycus alvarezii* (Doty.) Doty ex Silva (Solieriaceae) and *Corallopsis opuntia* J. Agardh (Hypneaceae) extracts have been observed to be more effective than *M. latifolia* extracts (Makkar and Chakraborty 2017). All the IC_50_ of the positive controls used in the study were compared with literature and the validity of the assays were supported (Mzid et al. 2017; Iheagwam et al. 2019a, 2020; Budipramana et al. 2019; Sarac et al. 2019). The present study showed that the isolated compounds **1**–**4** inhibited α-amylase and thus contributed to the α-amylase inhibition activity of the *M. latifolia* chloroform extract. Compound **2**, a furanocoumarin, was the most active α-amylase inhibitor among the compounds whereby its inhibition activity was significantly stronger than that of the positive control, acarbose. Other furanocoumarins such as imperatorin, bergapten, and psoralen have similarly demonstrated their respective antidiabetic efficacies through the inhibition of carbohydrate digesting enzymes (Bruni et al. 2019). In this study, compounds **1** and **4** showed considerable α-amylase inhibition. The results were in accordance with the findings from previous *in vitro* α-amylase inhibitory studies of these compounds (Hamid et al. 2015; Lin et al. 2016). In fact, phytosterols have been shown to greatly contribute to the potent α-amylase inhibition of certain algae species (Payghami et al. 2014). Despite their potential in inhibiting α-amylase, compounds **1**, **2**, and **4** were not effective against DPP-4. Only compound **3** showed mild DPP-4 inhibition activity. This study is the first to investigate the DPP-4 inhibition activities of compounds **1**–**4**. The observed differences in the compounds’ inhibition activity against different enzymes are most likely caused by the selective enzyme inhibition of the compounds (El Omari et al. 2019).

The active site of α-amylase is made up of three important residues: Asp197, Glu233, and Asp300. These residues work complementarily for the hydrolysis of substrates such as starch (Alqahtani et al. 2019). Other amino acid residues including Trp59, Trp58, and Tyr62 are important for substrate binding to the α-amylase enzyme (Ramasubbu et al. 2004). Acarbose which is a commercial inhibitor of α-amylase was found to interact with three critical residues along with other amino acid residues namely, Thr163, Tyr62, and His305 through hydrogen bonding with a binding affinity of −9.1 kcal/mol (Figure 2(a)). The extensive hydrogen bonding interactions with the enzyme were attributed to the numerous hydroxyl groups of acarbose. When compared to acarbose, compound **2** showed weaker binding affinity (−6.6 kcal/mol). The methoxy groups attached at the benzene moiety of compound **2** acted as hydrogen bond donors and formed hydrogen bonding interactions with the carboxyl group of Asp300 (catalytic triad residue), as well as the carbonyl groups of Tyr62 and Trp59. The hydroxyl group of residue Thr163 which acted as a hydrogen bond donor formed an additional hydrogen bonding interaction with the methoxy group of compound **2**. The aromatic system in **2** further stabilized the binding through hydrophobic π–π interactions with Trp58 and Trp59 (Figure 2(b)). These interactions were found to be consistent with the interactions of acarbose with α-amylase. This suggest that the presence of such interactions could be responsible for the inhibition of α-amylase. The binding affinity of compound **2** with α-amylase was lower than those previously reported for swermirin and methyl 3,4,5-trimethoxycinnamate in another study (Quek et al. 2020).

In contrast to acarbose and compound **2**, the binding affinity (−9.3 kcal/mol) of compound **1** and α-amylase was mainly established by vast hydrophobic interactions with Leu165, Tyr62, His299, His101, Leu162, Ile235, Ala198, His201, and Trp59 (Figure 2(c)). Due to the lack of hydrogen bond donor and acceptor in the structure, no hydrogen bonding interaction was observed between compound **1** and α-amylase. Interestingly, despite exhibiting stronger interactions (lower binding affinity) with amino acid residues of α-amylase compared to compound **2**, compound **1** exhibited lower experimental *in vitro* α-amylase inhibition. This finding could indicate that the interacting residues with compound **1** were considered less crucial for the inhibition of α-amylase. In the case of compounds **3** (−5.7 kcal/mol) and **4** (−6.0 kcal/mol), with almost similar binding affinities, the benzene moiety of the compounds and amino acid residue Tyr62 formed π–π hydrophobic contact. The oxygen atoms of the carboxyl group in compound **4** further accepted hydrogen bonds from the imidazole side chain of His299 and amino groups of Arg195 to form conventional hydrogen bonding interactions (Figure 2(d)). Meanwhile, the carboxyl group of Asp300 acted as a hydrogen bond acceptor while amino groups of His299 and Gln63 acted as hydrogen bond donors when interacting with compound **3** (Figure 2(e)). Compound **2** was an exception, in that both the hydrogen bonding and hydrophobic interactions contributed to the tightening binding between the compounds and α-amylase.

The active sites of DPP-4 include the amino acid residues Ser630, Asn710, His740, Glu205, Glu206, Tyr662, Ser209, Arg358, Phe357, Tyr547, Tyr631, Val656, Trp659, Tyr666, and Val711 (Kim et al. 2018). These critical amino acid residues were considered as the regions of interest of DPP-4 in which positive control, sitagliptin was found to form extensive interactions with these amino acids with a binding affinity of −8.6 kcal/mol. Hydrogen bonding interactions were observed between DPP-4 and sitagliptin through Arg358, Glu205, Glu206, Ser209, Arg125, and Tyr662 while hydrophobic interactions were observed with Ser630, Tyr666, and Phe357. The fluorine atoms in the structure further formed halogen bonding with residues Val207 and His740 (Figure 3(a)). For compound **3**, the best binding pose with a binding affinity of −5.7 kcal/mol showed that the aromatic system in the structure interacted with critical amino acid residues of DPP-4: Tyr666 and Tyr662 through a π–π bonding (Figure 3(b)). Tyr666 and Tyr662 were located near the catalytic triad (Ser630, Asn710, and His740) of the DPP-4 enzyme, therefore, interactions with Tyr666 and Tyr662 may interfere with the catalytic actions of the enzyme. The binding affinity of compound **3** with DPP-4 was lower than those reported for 1,2,4-benzenetriol, dodecanoic acid, and benzoic acid (Iheagwam et al. 2019b). Identical amino acid interactions have also been observed with other potent natural DPP-4 inhibitors such as flavones and resveratrol (Jha and Bhadoriya 2018).

From the *in silico* docking analysis between the active compounds and targeted enzymes, it was shown that the *in vitro* inhibitory properties of the compounds are strongly correlated to the extensiveness of their binding with critical amino acid residues of the enzymes. For instance, compound **2** which showed the highest *in vitro* α-amylase inhibition was found to form the most interactions with critical amino acid residues of the enzyme compared to other compounds. Compound **1**, despite showing better binding stability based on its lower binding affinity, interacted less with critical amino acids of α-amylase. This might explain its lower α-amylase inhibition activity compared to compound **2** and acarbose. Meanwhile, the coverage of DPP-4 active sites by compound **3** was lesser than that of sitagliptin which justifies its lower inhibitory activity against DPP-4. Thus, it is suggested that the binding with the critical amino acid residues of the enzymes play an important role in interrupting or retarding the enzyme activity.

Numerous studies have reported that antioxidant compounds such as swertianin, 1,2-dihydroxy-6-methoxyxanthone-8-*O*-β-d-xylopyranosyl, and α-hydroxy succinamic acid protects β-cells from reactive oxygen species (ROS)-induced damage and consequently prevent diabetes (Mahendran et al. 2014; Tanwar et al. 2017). Antioxidant compounds that occur in natural sources have thus garnered great attention and have been used to attenuate oxidative stress-induced diabetes (Unuofin and Lebelo 2020). In addition, oxidative stress has also been proven to contribute to the development of diabetes complications. DPPH is a free radical that is largely used to determine the general antioxidant or scavenging activity of plant extracts and compounds. The β-carotene bleaching assay has been a reliable method for measuring lipid peroxidation inhibitory potency of natural products. In the DPPH scavenging assay performed in this study, the absence or weak DPPH scavenging performance of the isolated constituents (**1–4**) compared to the positive controls could be explained by the lack of phenolic hydroxyl groups in their structures. Multiple hydroxyl groups of polyphenols play a substantial role in free radical-scavenging reactions (Kassim et al. 2019). The stronger DPPH free radical scavenging activity observed in compound **2** compared to other compounds could be due to the presence of methoxy groups, which increases its electron donating ability (Yeap et al. 2017). Compound **2** also showed good antioxidant activity in β-carotene bleaching, and can be explained by the ability of furanocoumarin to interact with the lipid phase of the linoleic emulsion, effectively enabling the scavenging of the radicals generated from linoleic acid (Jafari et al. 2016). The antioxidant properties of compound **2** in addition to its α-amylase inhibitory activity may enhance its potency as a blood glucose-lowering agent. The potency of α-amylase inhibition has been shown to be related to the presence of certain compounds such as tannins, phenols, flavonoids, and compounds with antioxidant activities (Jo et al. 2009; Asadi et al. 2011).

## Conclusions

This study identified the potential inhibitors of α-amylase and DPP-4 extracted from *M. latifolia*. The *in vitro* and *in silico* analysis revealed that halfordin (**2**) is comparable to the standard acarbose against α-amylase while methyl *p*-coumarate (**3**) despite only mildly inhibiting both α-amylase and DPP-4, could potentially be developed as a multimode antidiabetic agent. Halfordin (**2**) also showed potential as an antioxidant agent via the DPPH scavenging and β-carotene bleaching assay. Further pharmacological studies on the bioactive compounds are suggested. Phytochemical profiling of the active fractions should also be performed in the future to identify other bioactive compounds for the development of antidiabetic agents.

## Supplementary Material

Supplementary_material.docx
