# Supplementary material for: α-Amylase and dipeptidyl peptidase-4 (DPP-4) inhibitory effects of Melicope latifolia bark extracts and identification of bioactive constituents using in vitro and in silico approaches
Source: Pharm Biol. 2021 Aug 4;59(1):962–71. doi: 10.1080/13880209.2021.1948065 (PMC8344235; doi:10.1080/13880209.2021.1948065)
Supplement: Supplementary_material.docx [file IPHB_A_1948065_SM7905.docx]

**Supplemental Material**

**
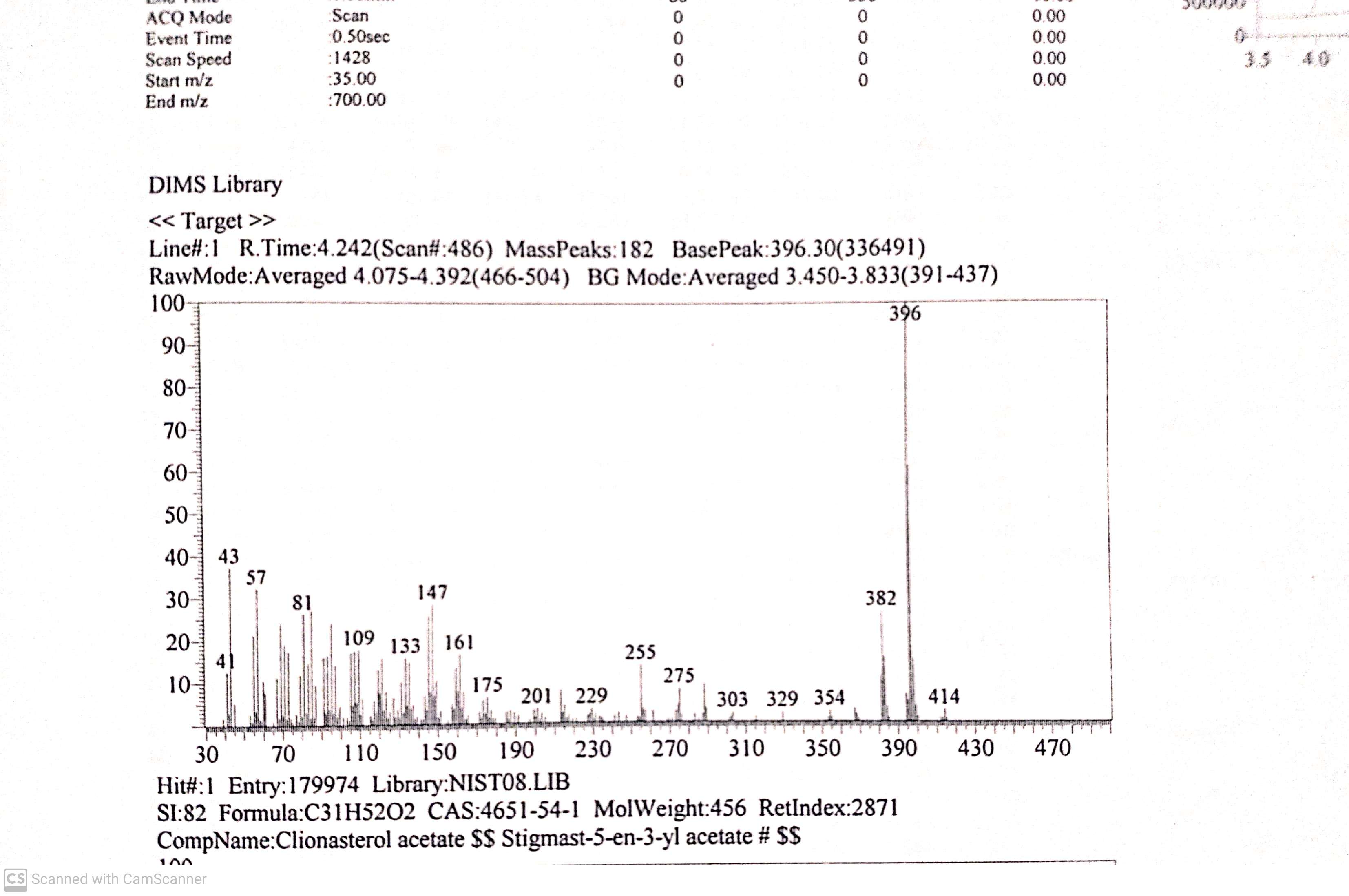
**

(a)


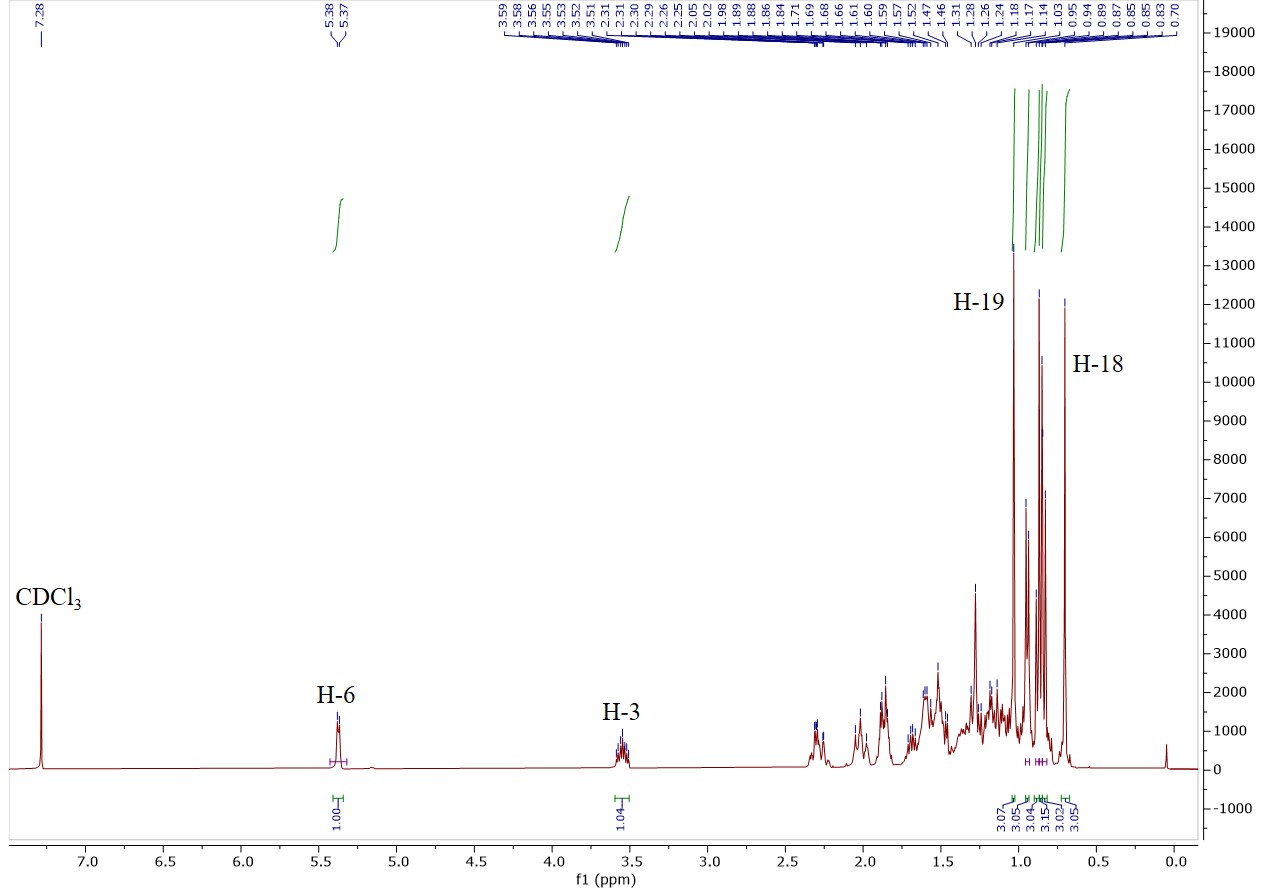


(b)


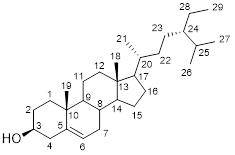

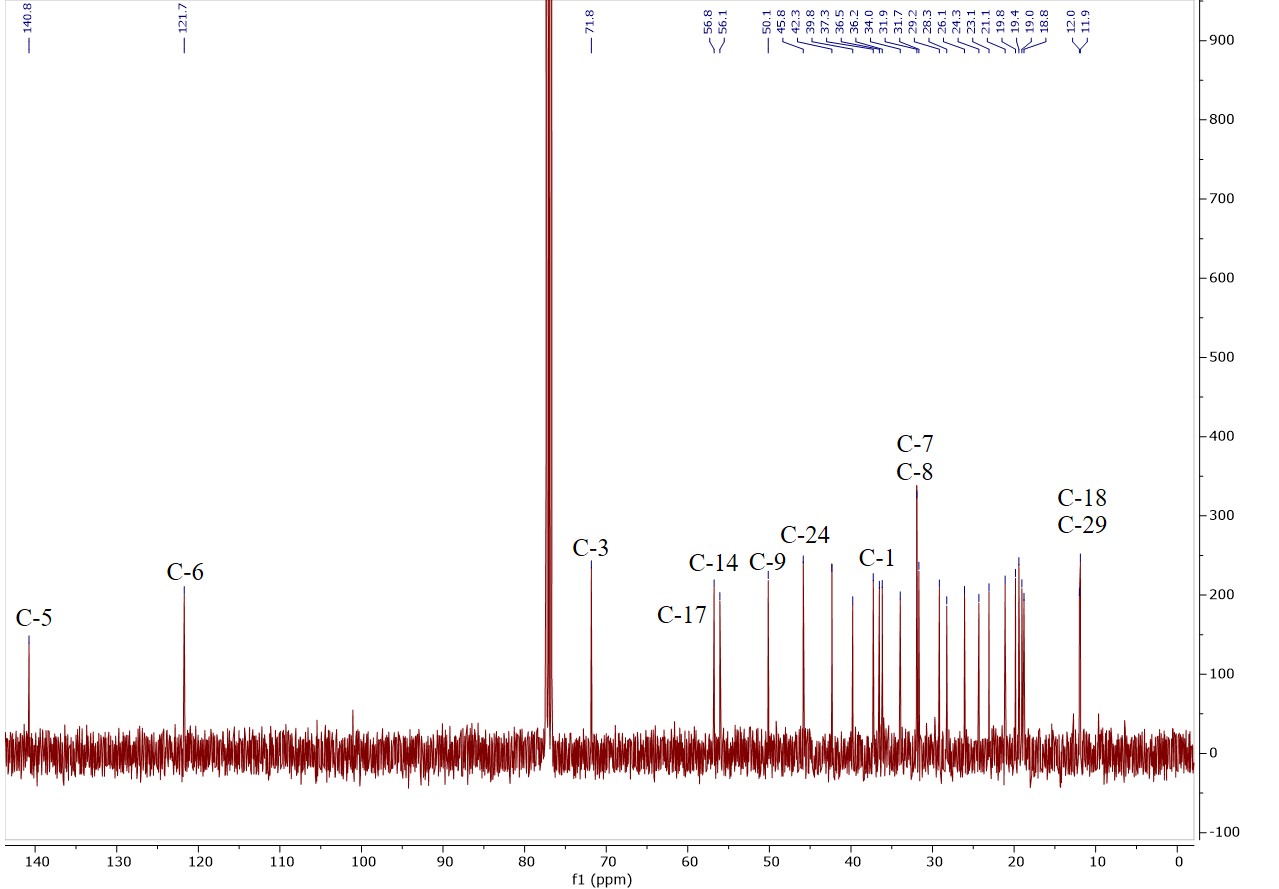


(c)

Supplemental Figure S1: (a) EI-MS (b) ^1^H NMR Spectrum (c) ^13^C NMR Spectrum of β-sitosterol, **1**.


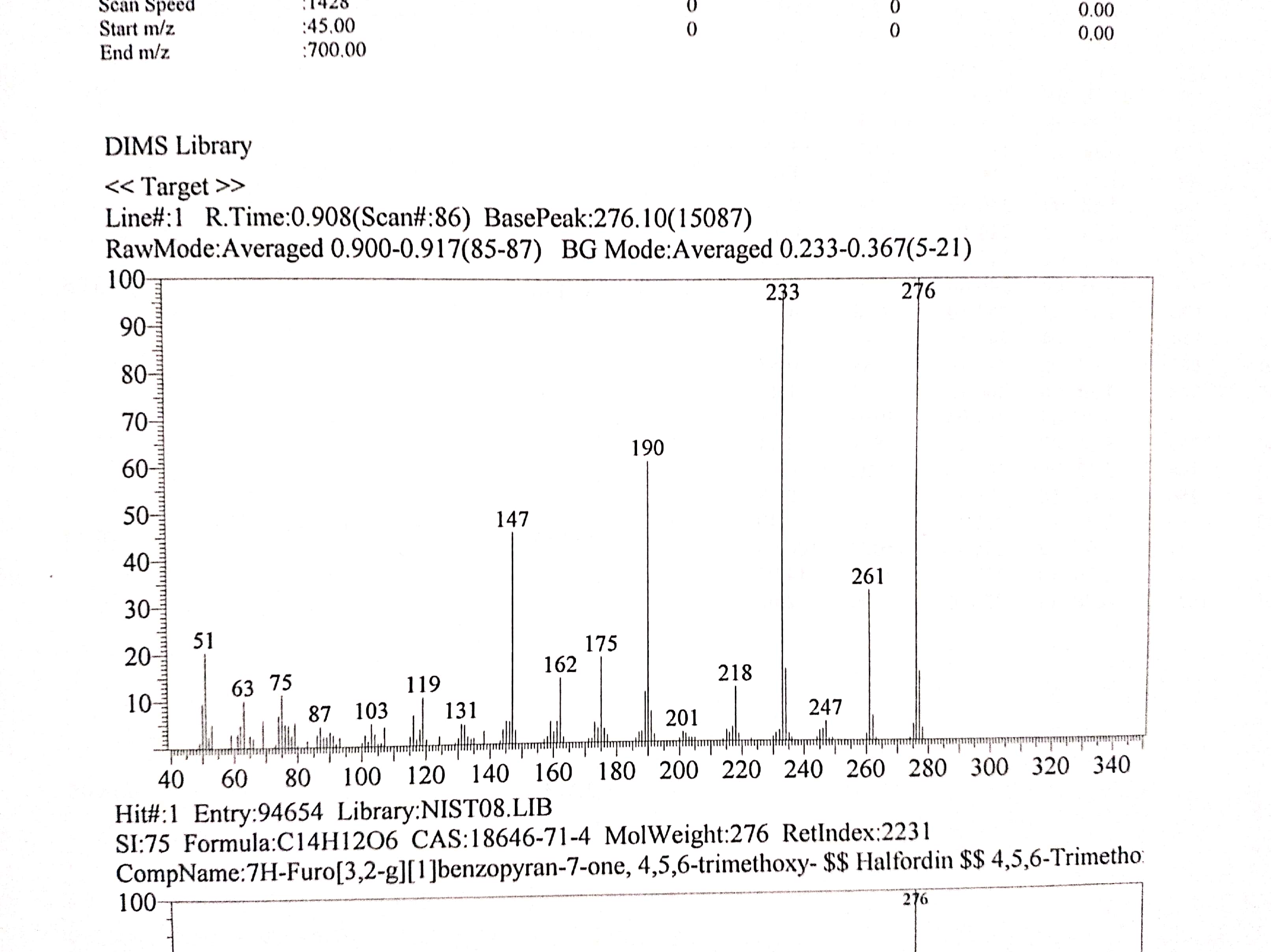


(a)


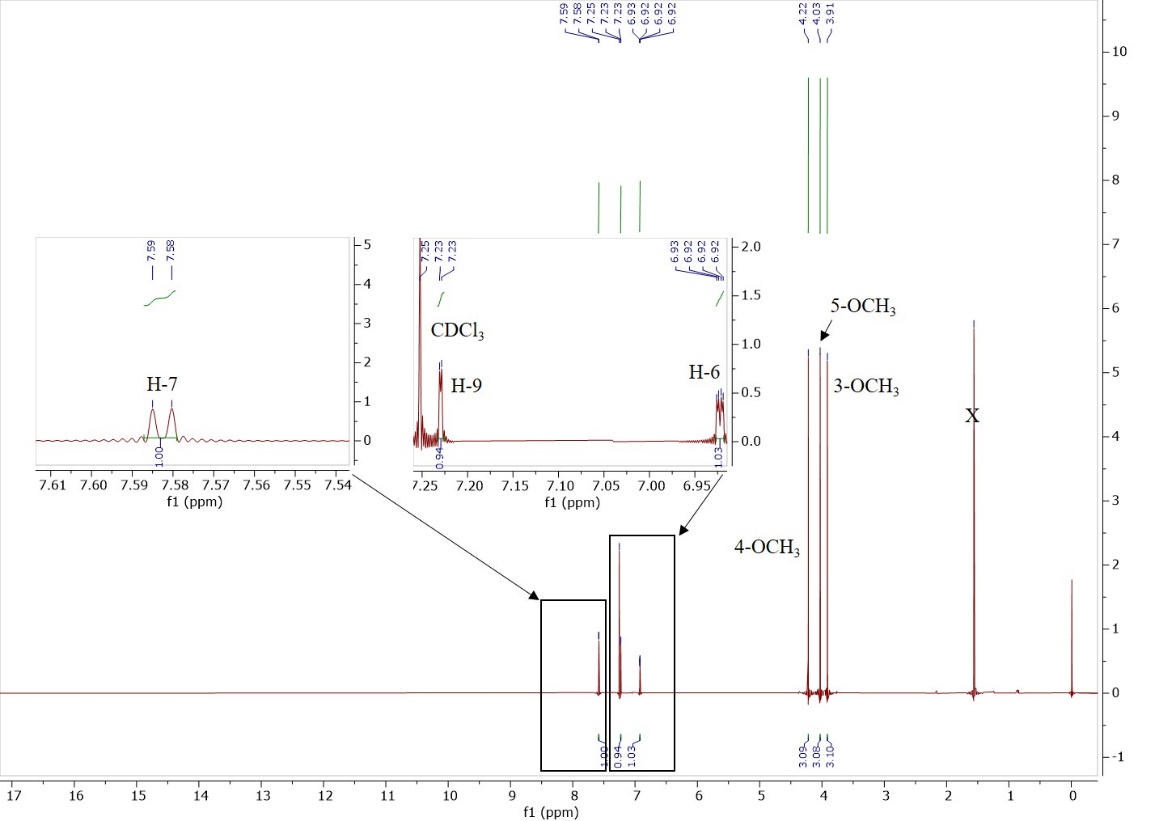


(b)


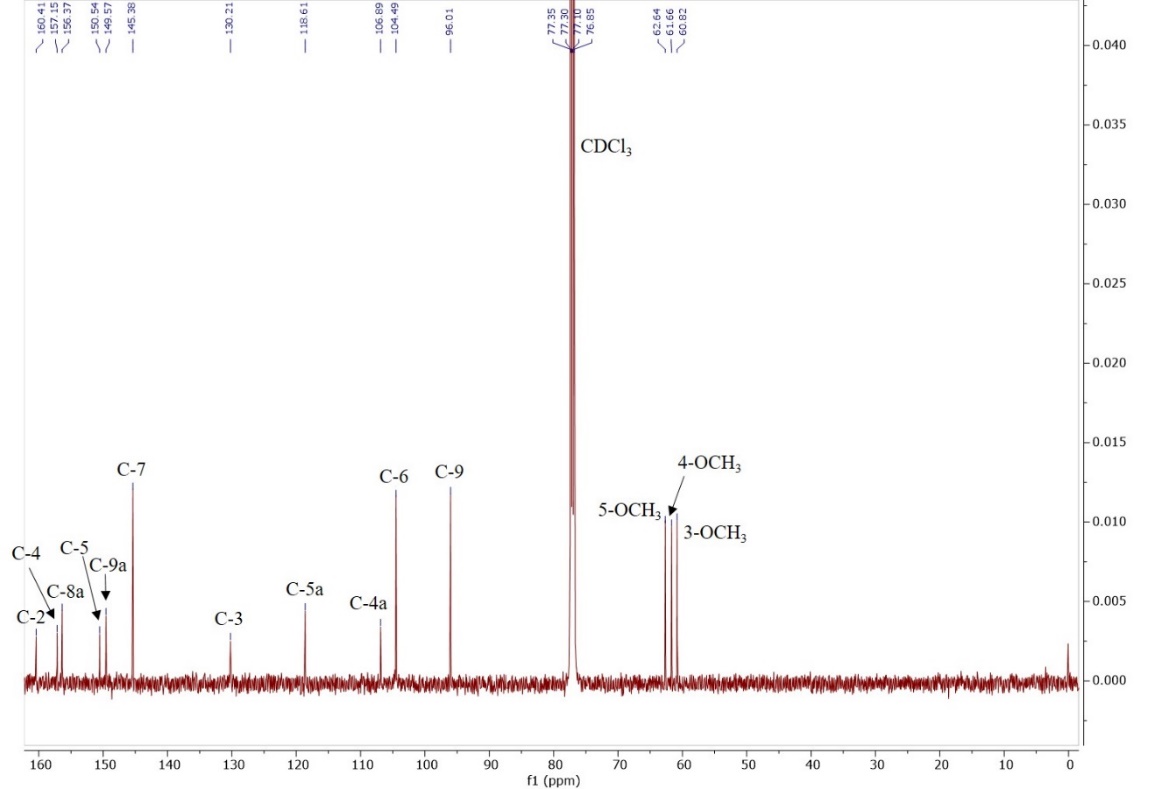


(c)

Supplemental Figure S2: (a) EI-MS spectrum (b) ^1^H NMR spectrum (c) ^13^C NMR spectrum of halfordin, **2**.


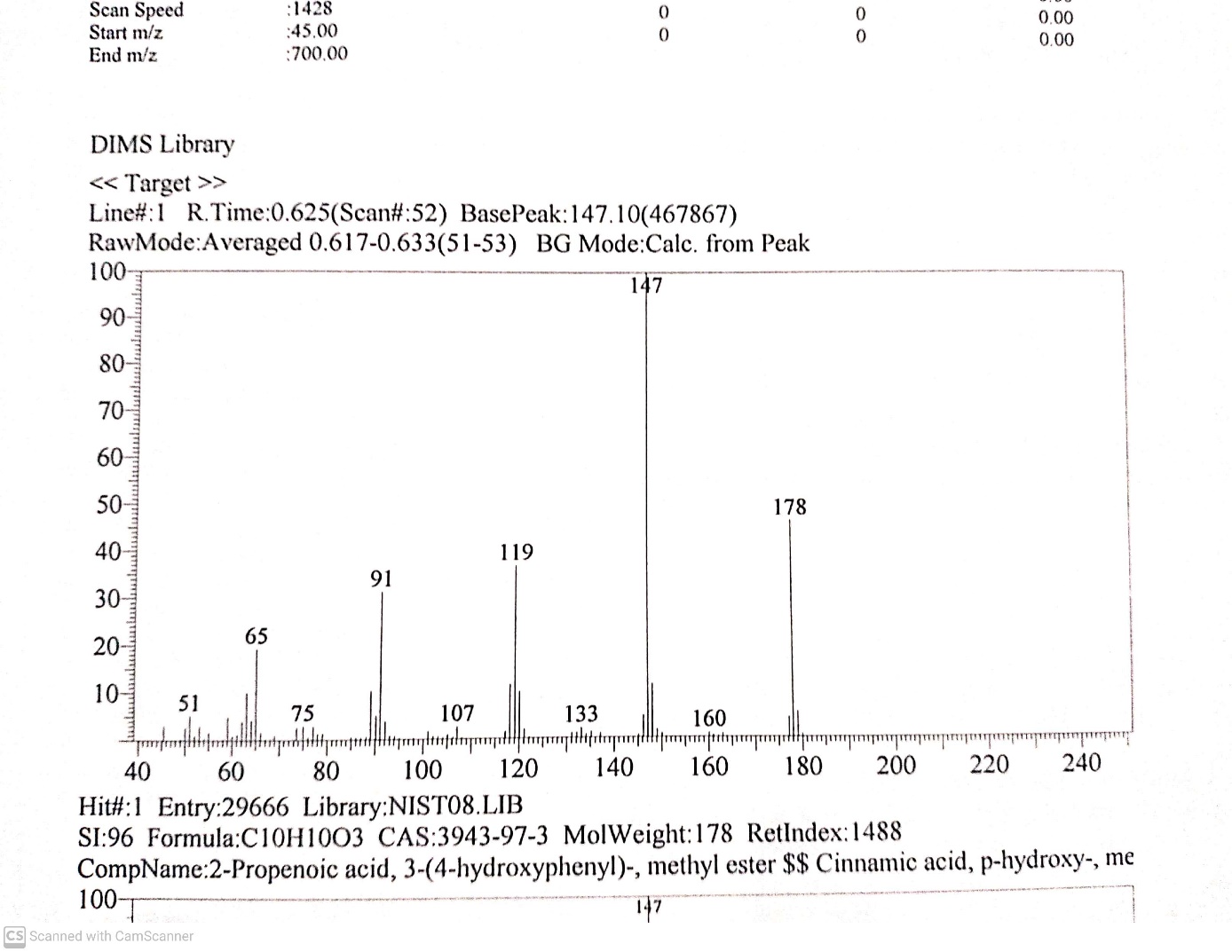


(a)


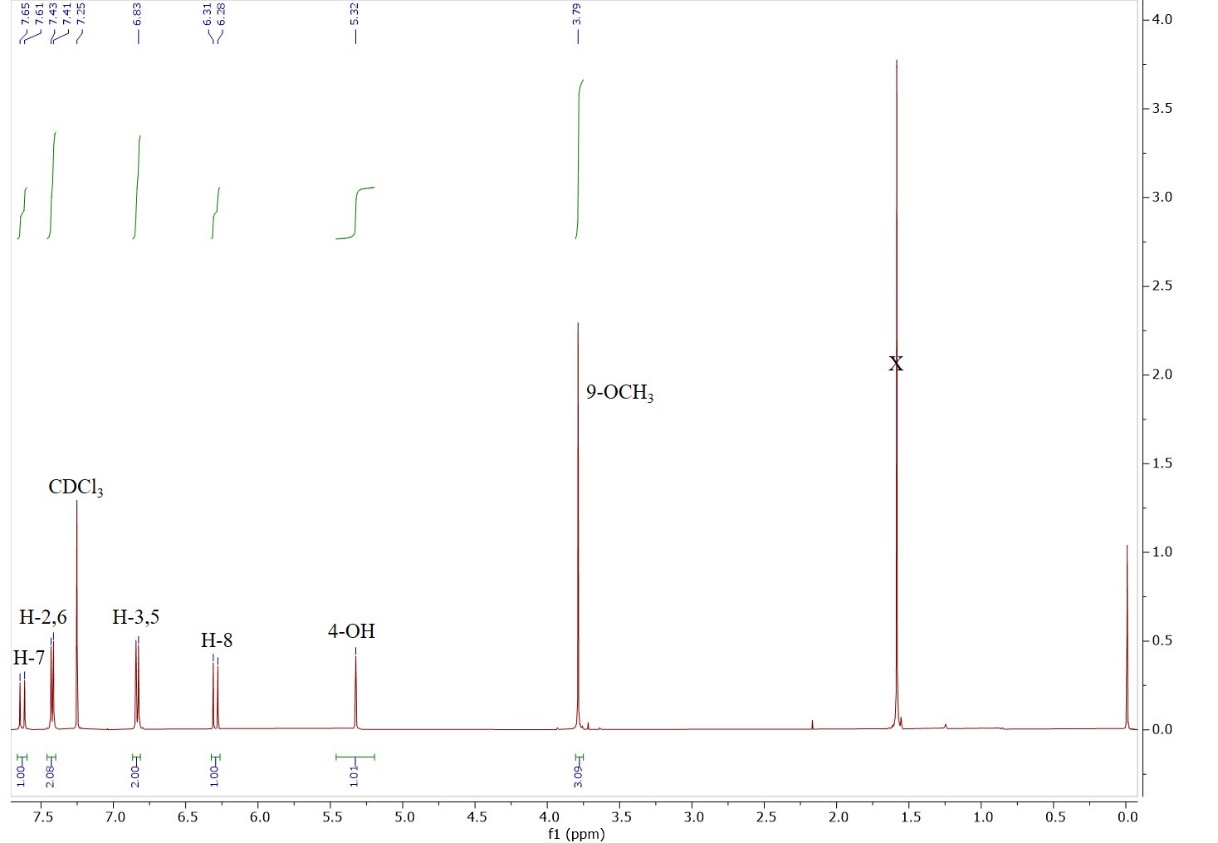


(b)


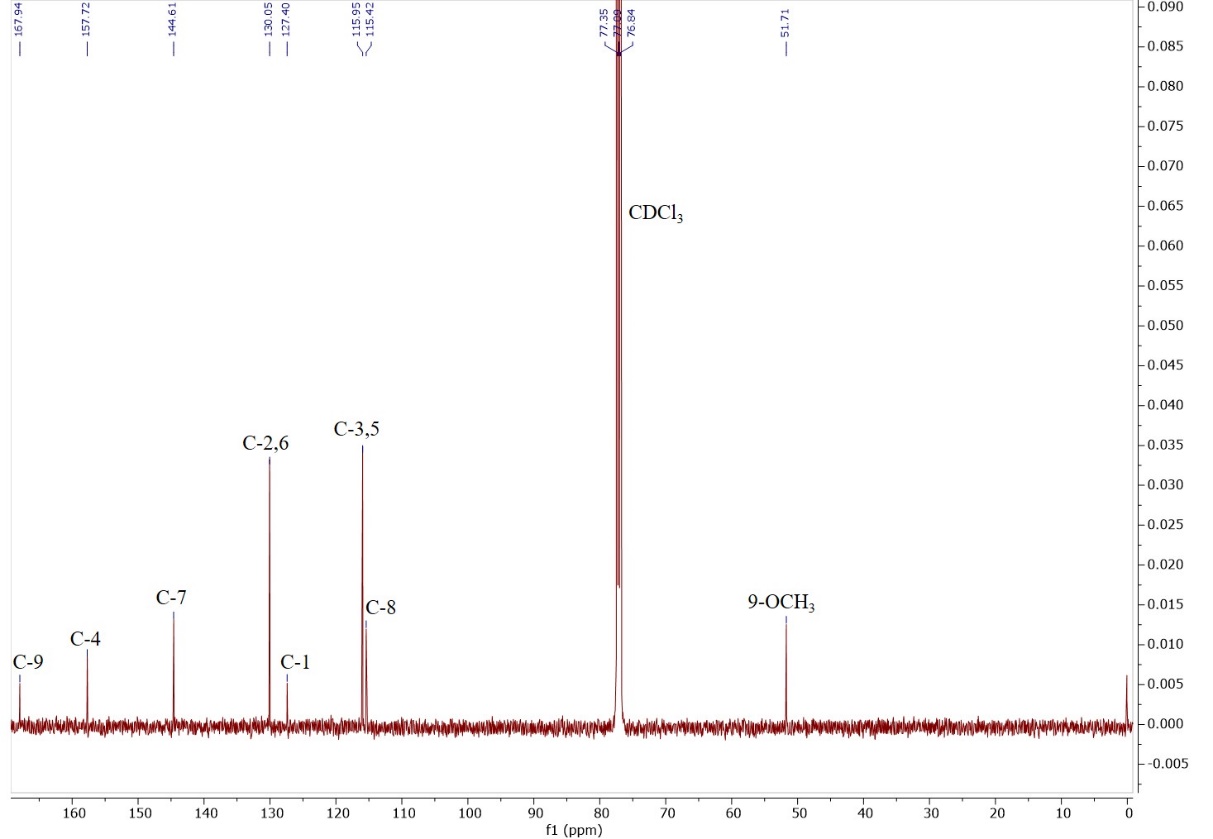


(c)

Supplemental Figure S3: (a) EI-MS spectrum (b) ^1^H NMR spectrum (c) ^13^C NMR spectrum of *trans*-methyl *p*-coumarate, **3**.


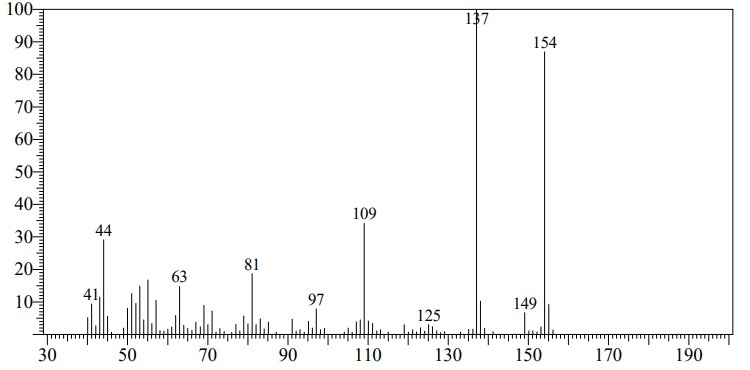


(a)


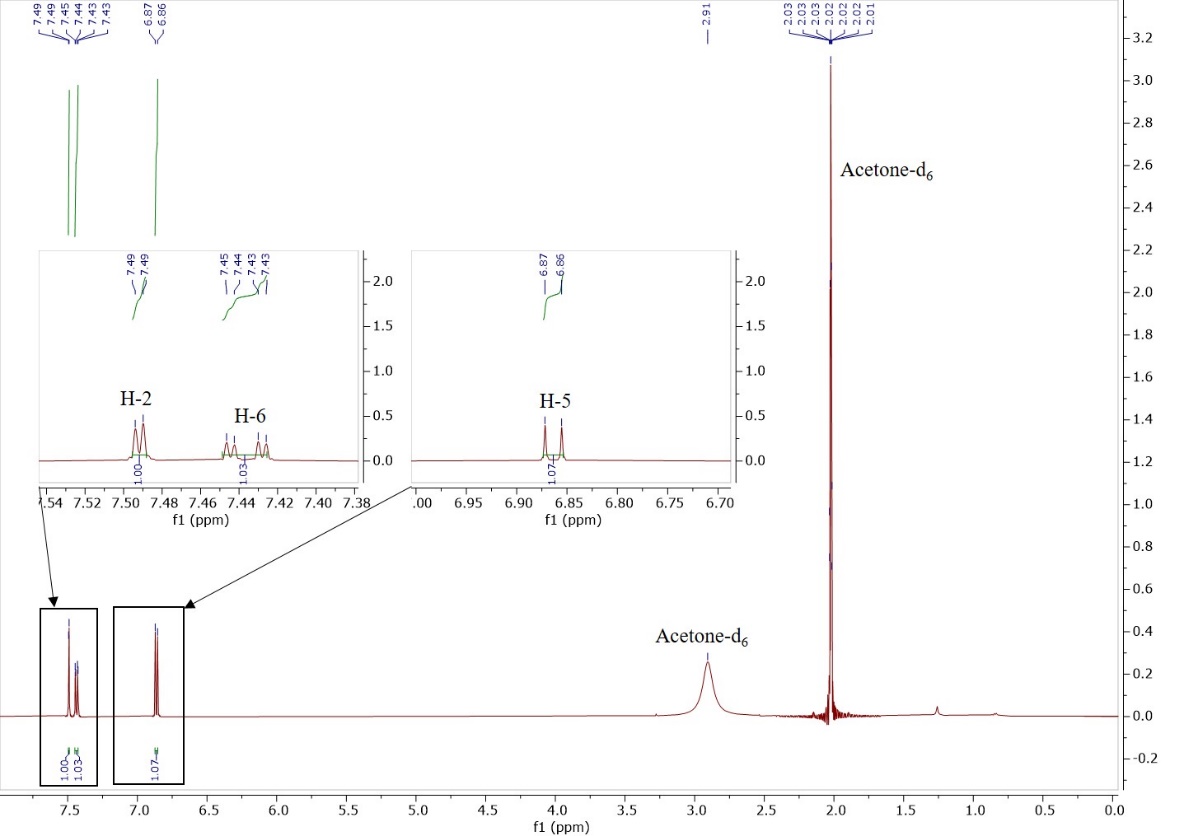


(b)


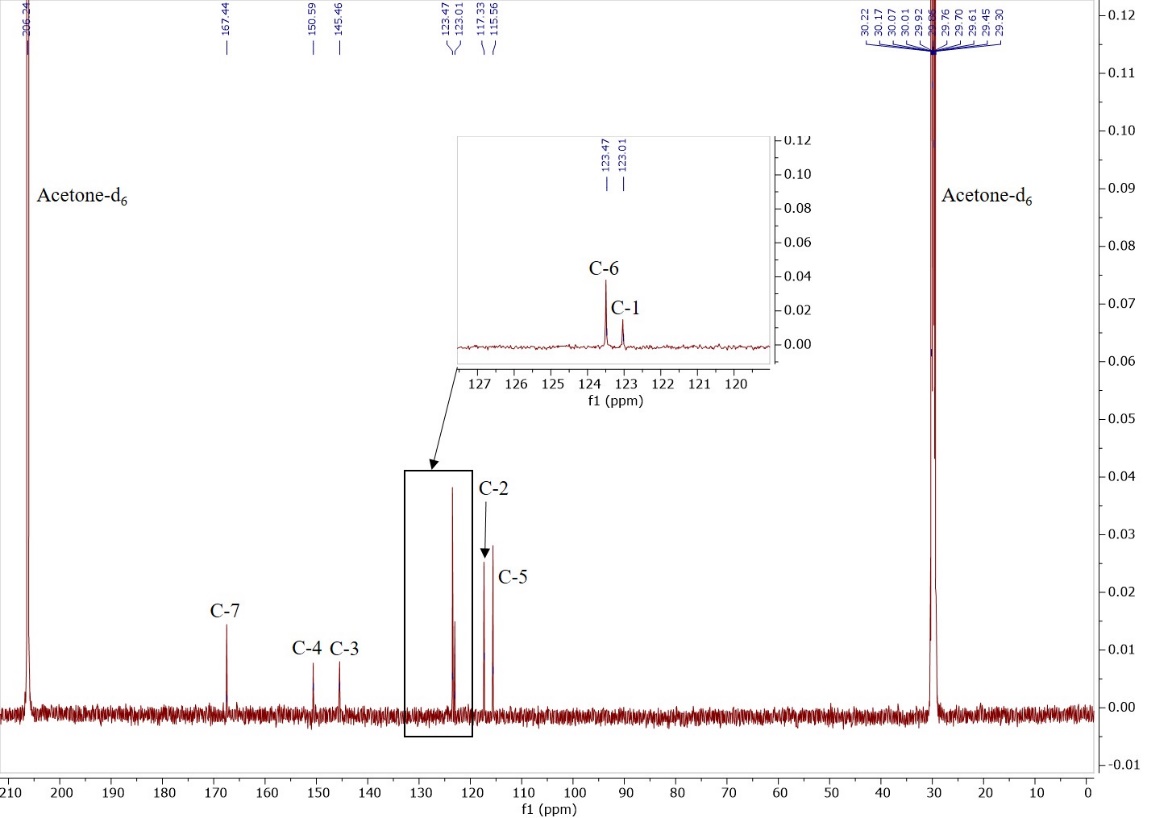


(c)

Supplemental Figure S4: (a) EI-MS spectrum (b) ^1^H NMR spectrum (c) ^13^C NMR spectrum of protocatechuic acid, **4**.

| 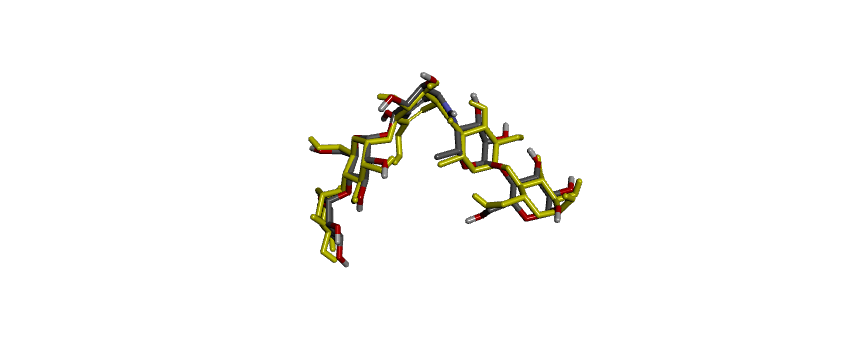 | 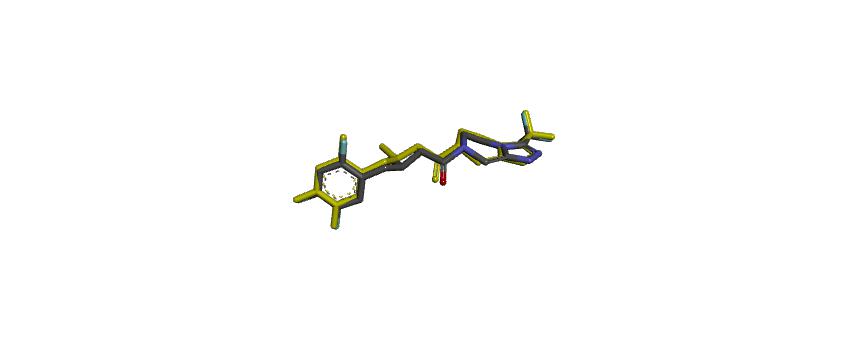 |
| --- | --- |
| (a) | (b) |

Supplemental Figure S5: Superimposed image of experimental pose and redocked pose (yellow) of co-crystallized (a) acarbose derived pentasaccharide and (b) sitagliptin.

| 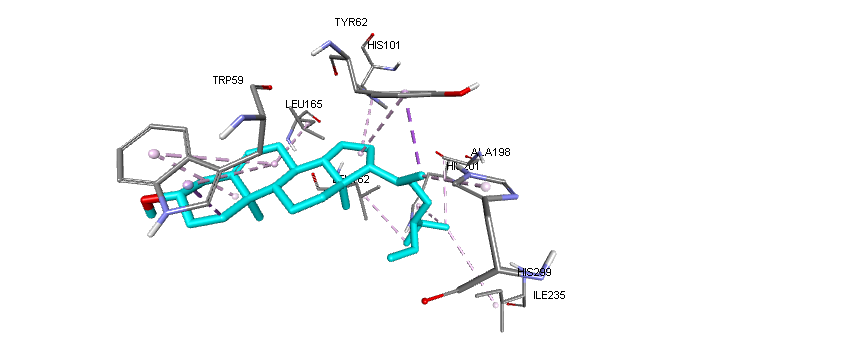 | 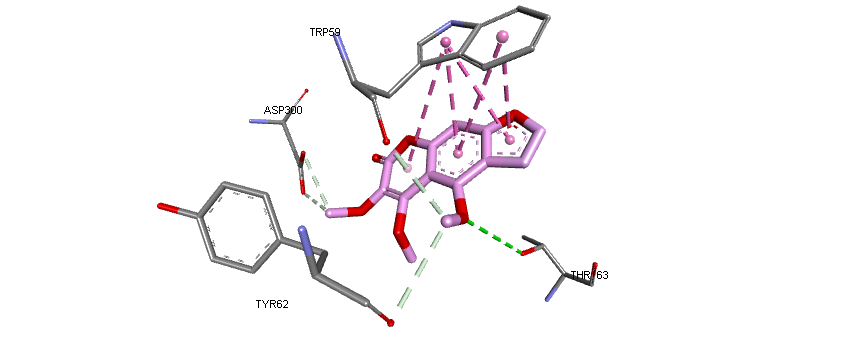 |
| --- | --- |
| (a) | (b) |
| 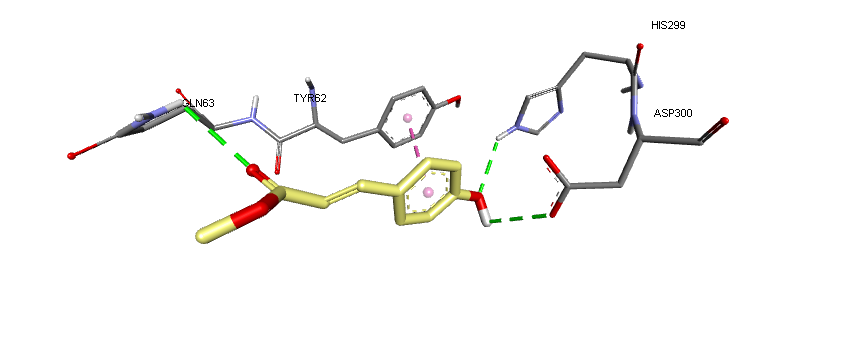 | |
| (c) | |
| 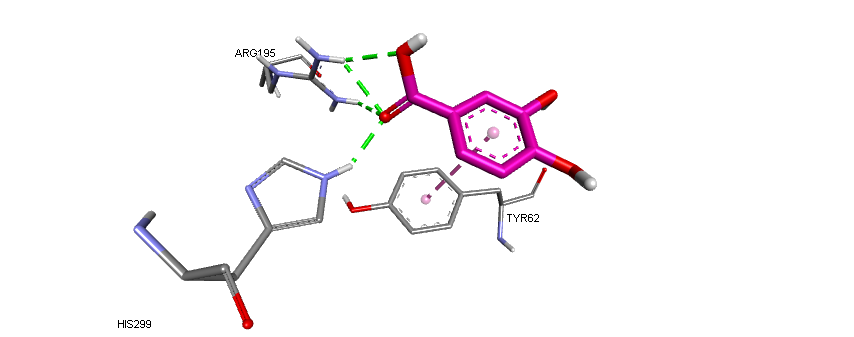 | 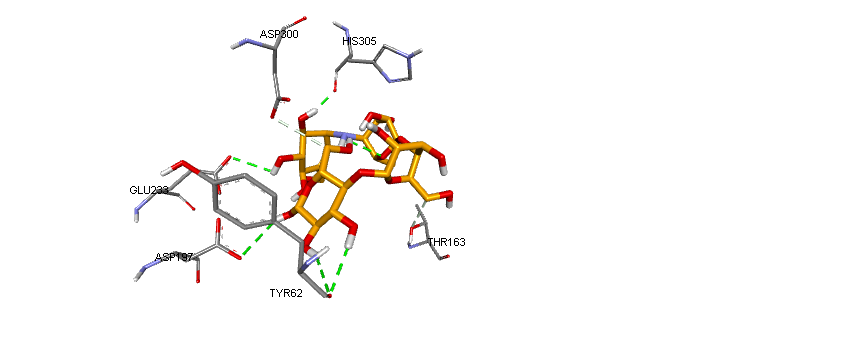 |
| (d) | (e) |

Supplemental Figure S6: Three-dimensional (3D) interaction diagram of the identified compounds with amino acid residues of α-amylase: (a) β-sitosterol, **1** (b) halfordin, **2** (c) methyl p-coumarate, **3** (d) protocatechuic acid, **4** (e) acarbose.

| 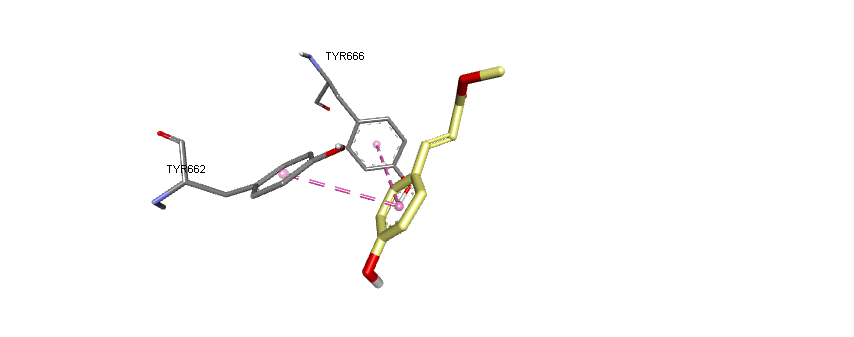 | 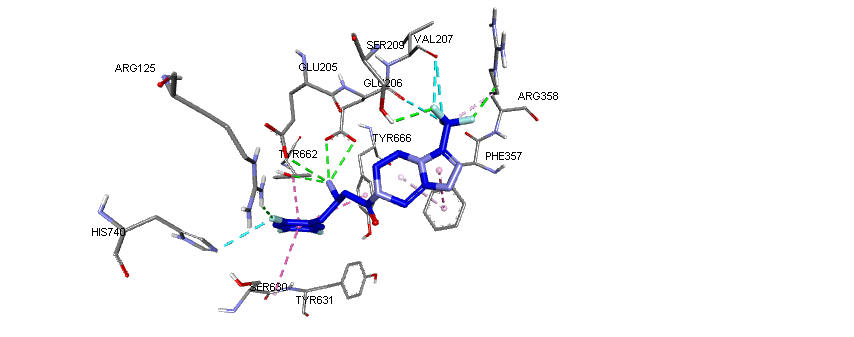 |
| --- | --- |
| (a) | (b) |

Supplemental Figure S7: Three-dimensional (3D) interaction diagram of the identified compound with amino acid residues of DPP-4: (a) methyl p-coumarate, **3** (b) sitagliptin.

Supplemental Table S1: Three-dimensional (3D) interactions of the identified compounds with amino acid residues of α-amylase.

| **Compound** | **Binding affinity (kcal/mol)** | **H-bond interaction** | | **Hydrophobic interaction** |
| --- | --- | --- | --- | --- |
|  |  | **H-bond donor** | **H-bond acceptor** |  |
| **1** | -9.3 | - | - | Leu165, Tyr62, His299, His101, Leu162, Ile235, Ala198, His201, Trp59 |
| **2** | -6.6 | Thr163 | Asp300, Trp59, Tyr62 | Trp59, Trp58 |
| **3** | -5.7 | Gln63, His299 | Asp300 | Tyr62 |
| **4** | -6.0 | Arg195, His299 | - | Tyr62 |
| **Acarbose** | -9.1 | - | Asp197, Tyr62, Glu233, His305, Asp300, Thr163 | - |

H-bond: Hydrogen bond

Supplemental Table S2: Three-dimensional (3D) interactions of the identified compound with amino acid residues of DPP-4.

| **Compound** | **Binding affinity (kcal/mol)** | **H-bond interaction** | | **Hydrophobic interaction** | **Halogen bond interaction** |
| --- | --- | --- | --- | --- | --- |
|  |  | **H-bond donor** | **H-bond acceptor** |  |  |
| **3** | -5.7 | - | - | Tyr662, Tyr666 | - |
| **Sitagliptin** | -8.6 | Arg125, Ser209, Arg358 | Glu205, Glu206, Tyr662 | Tyr662, Phe357, Tyr666, Ser630, Arg358, Phe357 | Glu206, Val207, His740 |

H-bond: Hydrogen bond
